# Erector Spinae Plane Block Decreases Pain and Opioid Consumption in Breast Surgery: Systematic Review

**DOI:** 10.1097/GOX.0000000000002525

**Published:** 2019-11-20

**Authors:** Hassan ElHawary, Kenzy Abdelhamid, Fanyi Meng, Jeffrey E. Janis

**Affiliations:** From the * Faculty of Medicine, McGill University, Montreal, Quebec, Canada; † Department of Plastic and Reconstructive Surgery McGill University Health Centre, Montreal, Quebec, Canada; ‡ Department of Plastic Surgery, Ohio State University Wexner Medical Center, Columbus, Ohio.

## Abstract

Adequate control of acute postoperative pain is crucial in breast surgeries, as it is a significant factor in the development of persistent chronic pain. Inadequate postoperative pain control increases length of hospital stays and risk of severe complications. Erector spinae plane block (ESPB) is a novel regional block that has the ability to sufficiently block unilateral multidermatomal sensation from T1 to L3. By reviewing the literature on ESPB, this paper aimed to elucidate its efficacy in breast surgery analgesia and its role in addressing the opioid crisis in North America.

**Methods:**

PUBMED, EMBASE, and Cochrane databases were systematically searched for relevant articles according to the Preferred Reporting Items for Systematic Reviews and Meta-analyses guidelines. Inclusion criteria included any articles that described ESPB in breast surgery. Exclusion criteria composed of articles that exclusively discussed other kinds of regional blocks.

**Results:**

Thirty-two articles including 6 randomized controlled trials were included in this review. ESPB demonstrated superior pain control and less opioid consumption compared with tumescent anesthesia or using no block. However, ESPB showed lower efficacy in pain control compared with pectoral nerve block. Patients experienced less nausea and vomiting and were overall more satisfied with ESPB compared with other pain control modalities. The vast majority of the studies reported the ease of ESPB administration, and only 1 case presented with a complication.

**Conclusions:**

ESPB is a promising form of regional anesthesia that can decrease postoperative pain and opioid consumption when used as part of multimodal pain analgesia for patients undergoing breast surgery.

## INTRODUCTION

Breast surgeries encompass a wide scope of procedures from breast enhancement to oncologic mastectomy and reconstruction. One common feature they share is the significant postoperative pain experienced by patients, with some studies showing that over 50% of patients undergoing mastectomy and reconstruction experience pain up to 1 year postoperatively.^1–3^ Although breast surgeries can significantly improve quality of life and be curative in cases of malignancy,^4,5^ acute and chronic postoperative pain can be severe and cause functional impairment.^6,7^ Although research in breast surgery is constantly evolving to provide better aesthetic outcomes with fewer complications,^8,9^ postoperative pain still remains a burden for patients and an unresolved challenge for surgeons.^10,11^

The past few decades have witnessed a surge in opioid consumption as a method of postoperative analgesia. Alarmingly, levels of addiction and opioid-related mortality have reached over 42,000 deaths annually in the United States alone.^12,13^ The opioid epidemic has fueled continuous efforts in improving pain management in plastic surgery procedures and specifically breast surgery.^14,15^ A case–control study of almost half a million individuals who underwent plastic or reconstructive surgery found that patients undergoing breast surgeries are the most susceptible to both acute and prolonged postoperative pain.^16^ Although many patients prefer tolerating pain rather than overconsuming opioids, the literature shows that breast surgeons usually overprescribe them.^17,18^ Therefore, it is the physician’s responsibility to seek benign pain control modalities to ease patients’ recovery and fight the opioid epidemic.

Although using regional blocks as an adjunct form of analgesia existed for many years, they have only recently increased in popularity as a method of postoperative pain management.^19,20^ This change parallels the surge in interest for quality improvement initiatives and Enhanced Recovery after Surgery pathways in hospitals to improve outcomes and increase patient satisfaction. A recent meta-analysis demonstrated superior pain control with regional blocks compared with opioid-based analgesia.^20^ In addition, they allow for earlier patient mobilization, faster return to function, and carry no risk of addiction and overdose.^21^

In surgeries for breast cancer specifically, there is further evidence that regional anesthesia attenuates the surgical response system and can reduce the progression of malignancy.^22^ Several regional nerve blocks have been proposed for breast analgesia.^23^ Popular examples include the pectoral nerve blocks (PECS I and II) and paravertebral blocks (PVBs).^24^ Although the efficacy of these blocks has been well demonstrated, they all incur limitations, such as increased hematoma rates, risk of pleural punctures, and intravascular injections.^25–27^ Furthermore, the spread of local anesthetic through the fascial planes in PECS can prevent the electrocautery from functioning at an acceptable level.^28^ A recent systematic review of fascial plane blocks in breast surgeries showed that none of the reviewed blocks provide complete analgesia to the whole breast region alone.^21^

The erector spinae plane block (ESPB) is a novel regional anesthesia technique first described by Forero et al in 2016 as a successful interfascial block for neuropathic pain in the thorax.^29^ It demonstrated the ability to sufficiently anesthetize unilateral multidermatomal sensation from T1 to L3 when administered at T5.^30^ When compared with the commonly used PVB in thoracotomies, ESPB showed similar pain relief results with less adverse effects.^31^ In the past 2 years, ESPB has been used in several breast surgeries, such as mastectomies and breast reconstruction.^32,33^ Although several systematic reviews summarize the efficacy and limitations of interfascial regional blocks, none have been conducted for ESPB in breast-related surgeries.

The goal of this study is to systematically review the literature on the efficacy of ESPB in breast surgeries, specifically, mastectomies, lumpectomies, breast augmentations, reductions, and reconstructions. By reviewing the literature, this paper aims to elucidate ESPB’s efficacy in breast surgery analgesia, and its potential role in addressing the opioid crisis in North America. It is hoped that this will encourage surgeons to comfortably prescribe less opioids and provide an alternative, safer pain management method for patients.

## METHODS

The PUBMED, EMBASE, and Cochrane databases were systematically searched for relevant articles using both keywords and MeSH terms. The specific search strategy used for PUBMED was the following: (“mastectomy” OR “lobectomy” OR “breast” OR “Breast”[Mesh] OR “reduction” OR “augmentation” OR “reconstruction”) AND (“erector”). Similar searches were conducted on the 2 other databases.

This systematic review was performed and reported in compliance with the Preferred Reporting Items for Systematic Reviews and Meta-analyses. Two authors independently reviewed all resulting search entries against the inclusion and exclusion criteria. Any disagreement over the eligibility of an article was adjudicated by an independent researcher. Inclusion criteria consisted of studies that described ESPB in adult females (>18 years) undergoing breast surgery. Due to the novelty of this technique, randomized controlled trials (RCTs), prospective studies, case control studies, and case series/reports were included. Furthermore, there was no lower limit on the number of cases within a study for it to be included. Exclusion criteria composed of articles that exclusively discussed other kinds of regional blocks, and those that discussed ESPB in nonbreast-related surgeries. Finally, commentaries and conference abstracts were excluded.

## RESULTS

**Fig. 1.**
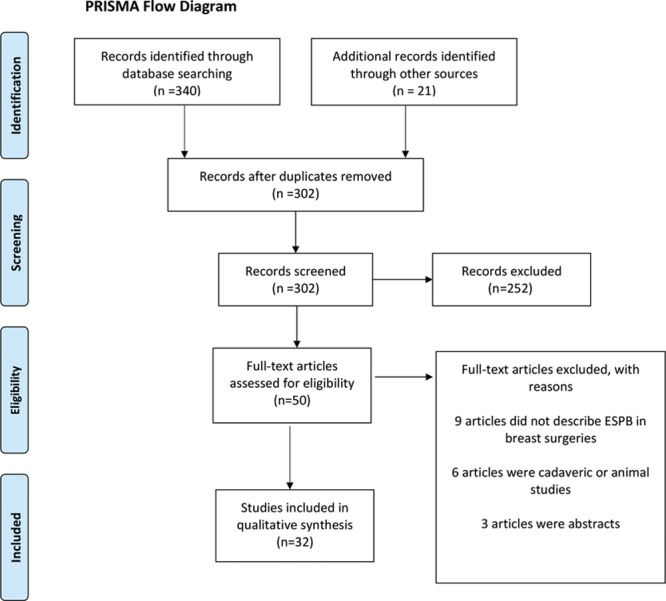
Search and screening process. PRISMA, Preferred Reporting Items for Systematic Reviews and Meta-analyses.

The primary search yielded 340 articles. Citations were manually checked, and 21 relevant citations were added to the pool of studies. Fifty-nine were excluded as duplicates. The remaining 302 studies’ titles and abstracts were independently assessed for inclusion/exclusion criteria, yielding a total of 50 articles. These articles were fully read, yielding a total of 32 articles to be included in this review.^32–52^ Out of the 32 articles, 6 were RCTs^34,40,45,51,53,54^ and 26 were case reports and case series. All of the articles were published between June 2017 and May 2019 (Fig. 1).

Two RCTs compared ESPB with controls (GA with no regional block),^33,40^ 2 compared ESPB with a PEC,^34,53^ 1 compared it with tumescent anesthesia,^45^ and the final RCT compared 2 groups receiving ESPB with different concentrations of bupivacaine.^51^ The sample sizes of the RCTs varied between 38 and 50 female patients of ages 18–70 years. Out of a total of 319 patients included in this review, 259 were participants of these RCTs.

Out of 26 case reports/series, 21 articles discussed the exclusive use of ESPB (continuous and noncontinuous),^32,33,35,36,39,41,42,44,46,47,49–51,55–57^ 5 discussed ESPB combined with another method of regional anesthesia [ESPB + PECS,^38^ ESPB + selective brachial plexus block,^39^ ESPB + transversus thoracic muscle plane block,^49^ ESPB + paravertebral nerve block (PVB),^49^ ESPB + rhomboid intercostal block + parasternal block^58^], and 1 discussed ESPB with the addition of a local anesthetic (liposomal bupivacaine).^43^ In total, the case reports and case series comprised 60 patients of ages 20–83 years. The reporting of outcomes was heterogeneous among the RCTs and the case reports/series. The most common measured outcome was postoperative opioid consumption (n = 23) followed by postoperative pain (n = 22).

### Postoperative Opioid Consumption

Postoperative opioid consumption was measured in all 6 RCTs. ESPB, compared with no block, was found to decrease opioid consumption within the first 24 hours post mastectomy as shown by 2 RCTs (*P* < 0.05).^40,54^ A higher concentration of bupivacaine was associated with less postoperative opioid consumption needed (*P* = 0.03).^51^ Furthermore, ESPB was found to significantly decrease opioid consumption within the first 24 hours post breast reduction when compared with tumescent anesthesia (*P* < 0.05).^45^ However, both Altiparmak et al and Gad et al showed that patients undergoing unilateral mastectomies who received an ESPB required significantly more tramadol and morphine compared with patients who received a PECS block (*P* = 0.001 and *P* < 0.001, respectively).^34,53^ However, it is important to note that both articles used more local anesthetic in the PECS block versus the ESPB (30 cc versus 20 cc) (Table 1).

**Table 1.** Opioid Consumption at 24 h Postoperatively as Measured in Included RCTs

References	No. Patients	Age of Patients	Type of Surgery	Groups	Opioid Consumption per Milligram at 24 h Postoperatively
Altiparmak et al^51,52^	41	38–70	Unilateral radical mastectomy with axillary lymph node dissection	(1)ESPB 0.375% bupivacaine (2)ESPB 0.25% bupivacaine	149.5 versus 199.5*
Altiparmak et al (2018)^34^	38	18–70	Unilateral radical mastectomy	(1)ESPB (2)PECS	196 versus 132.78*
Gad et al^53^	47	18–65	Unilateral modified radical mastectomy	(1)ESPB (2)PECS	16.7 versus 10.7*
Gurkan et al (2018)^40^	50	25–65	(1)Modified radical mastectomy (2)Simple mastectomy (3)Lumpectomy + sentinel lymph node biopsy (4)Lumpectomy + axillary dissection	(3)ESPB (4)No intervention	5.76 versus 16.6*
Oksuz et al (2018)^45^	43	18–70	Breast reduction surgery	(1)ESPB (2)Tumescent anesthesia	0.9 versus 2.09*
Singh et al^54^	40	20–55	Modified radical mastectomy	(1)ESPB (2)No intervention	1.95 versus 9.3*

* denotes statistically significant difference set at P<0.05.

The benefit of ESPB in reducing postoperative opioid consumption is further seen in the majority of case reports. Seventeen case reports/series measured patients’ postoperative opioid consumption. The majority of the patients required no postoperative opioids. No patient required any opioids after postoperative day 4. However, due to the heterogeneity of the opioid type used and the different routes of ingestion (PO versus IV), it is challenging to quantitatively compare postoperative opioid consumption in the different case series.

### Postoperative Pain Scores

Pain level was measured using either an NRS or a VAS in all 6 RCTs. When compared with no regional anesthesia (only GA), only 1 RCT found that patients who underwent an ESPB had significantly lower levels of pain at 0, 2, and 4 hours postoperatively (*P* < 0.05),^54^ whereas the other RCT evidenced no differences in pain between the patients who received ESPB and those who received no regional anesthesia.^40^ Furthermore, Oksuz et al found that patients undergoing ESPB had significantly less postoperative pain compared with tumescent anesthesia (*P* < 0.0001).^45^ The RCT comparing different concentration of bupivacaine in ESPB showed that patients who received the higher concentration of 0.375% had significantly less pain levels at every measured time point (*P* < 0.05).^51^ Finally, the 2 RCTs that compared ESPB with a PECS block found that patients undergoing the former block had higher levels of pain in the first 24 hours postoperatively (*P* < 0.05).^34,53^ Pain scores were further mentioned in 16 case reports, most of which (15 out of 16) reported the mean pain scores to be less than 5 on the VAS or NRS during the first 24 hours postoperatively. The maximum pain score reported by 1 patient was 6.^43^ Postoperative rescue analgesia was provided to patients with an NRS of 4 or higher.

### Postoperative Symptoms of Nausea/Vomiting

Symptoms of nausea and vomiting were measured in 4 of the 6 RCTs. Patients who received ESPB were found to have significantly less complaints of postoperative symptoms of nausea/vomiting (PONV) when compared with those who received tumescent anesthesia (*P* < 0.005).^45^ Although there was no significant difference between patients who received ESPB and those who received no regional anesthesia (*P* = 0.768) in 1 of the studies,^40^ another showed that 5 of 20 control patients in comparison with none of the ESPB patients required rescue metoclopramide for severe PONV.^54^ Furthermore, a higher concentration of bupivacaine was not shown to affect PONV, as demonstrated by Altiparmak et al^51^ One case series on 2 patients measured PONV and simply stated that no PONV was observed.^59^

### Intraoperative Opioid Requirement

Intraoperative opioid requirement due to pain response was measured in 2 of the 6 RCTs. The mean fentanyl requirement was found to be similar in both groups that were administered ESPB with different bupivacaine concentrations (*P* = 0.289).^51^ Furthermore, Gad et al found that there was no difference in the mean intraoperative fentanyl requirements between patients who underwent an ESPB compared with a PECS.^53^ Only 5 case series/reports discussed the use of intraoperative opioid requirement for pain response.^34,46^ Talawar et al found that 6 of 10 patients receiving ESPB required additional intraoperative fentanyl.^46^ Two other case reports mentioned the use of 150^58^ and 100 μg^60^ of fentanyl intraoperatively. On the other hand, Altiparmak et al and Kim et al reported that no additional intraoperative opioids were required for patients on ESPB.^34,61^

### Other Outcomes

The other outcomes reported include patient satisfaction, hospital length of stay, quality of sleep, and cortisol and prolactin levels. Two RCTs reported patient satisfaction.^45,54^ Patient satisfaction was reported to be better in the ESPB group compared with the control group of no regional anesthesia (*P* < 0.0001), and the tumescent anesthesia group (*P* < 0.001). Furthermore, 1 of the case reports mentioned patient satisfaction by stating that the patient was overall satisfied with the block. The case report failed to mention what test was used to measure satisfaction.^61^ The lengths of hospital stay of only 4 patients were reported, all of which varied between 8 hours and 3 days. However, due to the heterogeneity of the breast surgeries performed, no meaningful conclusion about length of hospital stay associated with ESPB should be drawn.^35–37,61^ Only 1 case report measured patient sleep quality and mentioned no postoperative insomnia.^50^ Prolactin and cortisol levels were measured in only 1 RCT which found no difference in either levels between patients who underwent ESPB and those who underwent a PECS block. However, both groups evidenced a significant decrease in both hormones 24 hours postoperatively.^53^

### Complications of ESPB

Only 2 articles (3 cases in total) reported complications. Ueshima and Otake reported 2 cases where there was inadequate intraoperative analgesic effect of the ESBP on T2–T6 intercostal nerves when assessed 20-minute postblock administration.^48^ Only 1 major complication was recorded in a patient with a pneumothorax 3 minutes after the administration of ESPB.^62^

## DISCUSSION

The present systematic review elucidates the potential benefits of ESPB in breast surgeries. All of the RCTs demonstrated positive efficacy of ESPB. As an adjunct to general anesthesia, it is found to be superior in decreasing postoperative pain and opioid consumption in patients undergoing mastectomies. When compared with tumescent anesthesia, ESPB was associated with less postoperative pain, opioid consumption, and overall greater patient satisfaction post breast reduction.^45^ Although PECS block was superior to ESPB block in terms of postoperative pain and opioid consumption, the authors of this review believe that ESPB is still a suitable option for breast surgeries especially due to the ease and safety of administration and the higher risk of complications associated with PECS blocks.^34,53^

Furthermore, different concentrations of local anesthetic agents, along with differences in patient positioning, can have varied clinical effects. Administration of ESPB using a higher concentration of local anesthetic can lead to stronger effects, as seen by Altiparmak et al, where 0.375%, compared with 0.25% of bupivacaine, had a superior outcome in reducing postoperative opioid consumption. Moreover, the position of the patient is known to affect the diffusion of the anesthetic agent. Although Forero et al originally described the technique to be administered whereas the patient is seated,^29^ Ueshima and Otake showed that the block still provided adequate breast analgesia when administrated in the lateral decubitus position, which allows the patient to be under general anesthesia before administration of the block.^49^ Aygun et al further demonstrated successful administration of ESPB via the “Dry Leaf technique” that allows the patient to be in the supine position. This permits administration of the block after commencement of the surgery.^63^ All 32 articles included in this review performed ESPB as described by Forero et al, but with slight modifications to the technique regarding position of patient (sitting/lateral decubitus/prone/supine), spine level of injection (T2–T5), and local anesthetic used (Table 2).

**Table 2.** ESPB Technique as Performed in Included Articles

Patient Position	References	Injection Level	Anesthetic
Sitting	Finneran IV et al (2017)^39^	T3	20-mL 0.5% ropivacaine with 2.5-μg/mL epinephrine
		T2 and T4	15-mL 0.5% ropivacaine with 2.5-μg/mL epinephrine
	De Cassai et al^38^	T4	20-mL 0.5% ropivacaine with epinephrine
	Altiparmak et al^51,52^		20-mL 0.375% bupivacaine versus
			20-mL 0.25% bupivacaine
	Oksuz et al (2018)^45^		20-mL 0.25% bupivacaine
	Nair et al^44^		30-mL 0.25% bupivacaine
	Ueshima and Otake^48^	T5	25-mL 0.25% levobupivacaine
	De Cassai et al^38^		20-mL 0.5% ropivacaine
	Singh et al^33^		25-mL 0.25% bupivacaine
	Singh et al^54^		20-mL 0.5% bupivacaine
	Talawar et al (2018)^46^		20-mL 0.375% ropivacaine
	Altiparmak et al (2018)^34^		25-mL 0.25% bupivacaine
	Bonvicini et al^36^		23-mL (75-mg ropivacaine and 16-mg mepivacaine)
	Kumar et al^43^		25-mL 0.25% bupivacaine and 266-mg liposomal bupivacaine
	Veiga et al^50^		20-mL 0.5% levobupivacaine
	Bonvicini et al^35^		25-mL (75-mg ropivacaine and 20-mg mepivacaine)
Lateral decubitus	Gad et al^53^	T4	20-mL 0.25% levobupivacaine + 0.5-μg/kg dexmedetomidine
	Kim et al^61^		20-mL 0.5% ropivacaine with epinephrine Postoperative infusion: 0.375% ropivacaine with epinephrine at 20 mL/8 h for 48 h
	Ueshima^62^		20-mL 0.25% levobupivacaine
	Ueshima^59^		Single injection, bilateral infusion: 20-mL 0.25% levobupivacaine per side
	Selvi and Tulgar^60^		20-mL mixture of 10-mL 0.25% bupivacaine + 5-mL 0.5% lidocaine per side
	Kimachi et al^42^	T5	20-mL 0.5% ropivacaine with epinephrine and 8-mg dexamethasone
	Kwon et al^56^		30-mL 0.375% ropivacaine with epinephrine Postoperative infusion: bolus of 30-mL 0.375% ropivacaine with epinephrine every 12 h for 48 h
	Altiparmak et al (2018)^34^		20-mL 0.25% bupivacaine
	Jain et al^41^		20-mL 0.25% bupivacaine Postoperative infusion: 0.25% bupivacaine at 5-mL/h for 72 h
	Ohgoshi et al^32^		20-mL 0.375% ropivacaine Postoperative infusion: 0.2% ropivacaine at 8 mL/h
	Tanaka et al^47^		20-mL 0.375% levobupivacaine Postoperative infusion: 0.1% levobupivacaine at 20 mL/8 h for 48 h
	Ueshima and Otake^49^		30-mL 0.25% levobupivacaine
	Orozco et al^57^	T6	20-mL mixture of 0.25% bupivacaine with epinephrine + 0.5% lidocaine
Prone	Gurkan et al (2018)^40^	T4	20-mL 0.25% bupivacaine
Supine	Aygun et al^63^	T3/T4	30-mL mixture of 15-mL 0.5% bupivacaine + 7.5-mL 2% lidocaine + 7.5-mL normal saline

Adequate regional block anesthesia can potentially allow patients to avoid general anesthesia which would decrease perioperative complications.^64^ In fact, several case reports showed successful breast operations using ESPB with sedation, without the use of general anesthesia.^37,38,42,61^ This is very promising as it allows high-risk patients with cardiac comorbidities and older patients to undergo breast surgeries whereas avoiding potential complications associated with general anesthesia.

A major limitation to the use of any regional block in breast surgery is the complex innervation of the region and the potential for block failure. The vast majority of the studies did not assess the sensory analgesia via pinprick/ice sensation testing, and therefore potential failures were left undetected. Four studies reported the need for intraoperative fentanyl use due to augmented pain response which could indicate inadequacy of ESPB.^46,51,58,60^ One case series reported 2 patients for which the block failed and an adjunctive block had to be administered.^48^ Several reports opted to combine ESPB with another block to achieve complete anesthesia of the breast and axilla region. Another limitation of regional blocks, including ESPB is their duration of action, which is limited to 12–24 hours with traditional local anesthetics.^43^ However, Kumar et al was able to prolong the analgesic effect of ESPB to over 72 hours with the addition of liposomal bupivaine.^43^

ESPB is an easy-to-administer block because of the simple identification of anatomic landmarks on ultrasound^65^ and is relatively safe due to the lack of vital structures in the immediate vicinity that are at risk of needle injury.^41^ Except for 1 case report where the patient developed a pneumothorax secondary to ESPB,^62^ there have been no major complications associated with the block according to this systematic review, and other reviews on regional anesthesia.^66^ As part of a multimodal pain analgesia, ESPB in breast surgery can help provide more effective perioperative pain management, and as such reduce postoperative pain and opioid requirement, leading to enhanced recovery and a shorter length of hospital stay. Finally, this technique may allow breast surgeries to be performed in an ambulatory setting, without the use of general anesthesia.^67^

Although the results of this review are promising, it has several limitations. The main one is the lack of quantitative analysis, which is due to the limited number of RCTs and the heterogeneity of the breast procedures performed, and the outcomes measured. This review included different study types such as case series and RCTs which hinder the level of evidence and conclusions of this review. The majority of RCTs excluded patients with ASA score of more than 2, and given the importance of avoiding general anesthesia and reducing opioid consumption in higher risk patients (ASA>2), the authors of this study decided to include case reports/series to more accurately report the benefits of ESPB in these patient populations.

Future studies should aim to investigate the variables that impact the spread of ESPB, such as patient position, age, and body habitus. In addition, future studies should aim to confirm whether ESPB provides adequate analgesia to the whole breast region via pinprick sensation testing. Furthermore, more RCTs should be conducted to verify the efficacy of ESPB compared with other widely used regional blocks, such as PVB and serratus anterior block. Future studies should also conduct cost–benefit analyses of ESPB which would provide great incentive in setting guidelines for breast perioperative pain control. Finally, none of the reviewed studies measure long-term opioid use.

## CONCLUSIONS

Given the prevalence of breast surgery, its risk for chronic postoperative pain, and the prolonged postoperative opioid use, there is growing interest for improved perioperative pain control using regional anesthesia.^16,23^ The ESPB is a promising form of regional anesthesia that can decrease postoperative pain and opioid consumption when used as part of multimodal pain analgesia. The technique is easy to perform under ultrasound guidance with a very low rate of complications.
